# Tapered Polymer Fiber Sensors for Reinforced Concrete Beam Vibration Detection

**DOI:** 10.3390/s16122149

**Published:** 2016-12-16

**Authors:** Dong Luo, Zainah Ibrahim, Jianxun Ma, Zubaidah Ismail, David Thomas Iseley

**Affiliations:** 1 School of Human Settlements and Civil Engineering, Xi’an JiaoTong University, Xi’an 710049, China; majx@mail.xjtu.edu.cn; 2 Department of Civil Engineering, Faculty of Engineering, University of Malaya, Kuala Lumpur 50603, Malaysia; zainah@um.edu.my (Z.I.); zu_ismail@um.edu.my (Z.I.); 3 Director of Trenchless Technology Center (TTC), Louisiana Tech University, 599 Dan Reneau Dr., Engineering Annex, Ruston, LA 71272, USA; dtiseley@latech.edu

**Keywords:** tapered polymer fiber sensor, accelerometers, vibration, natural frequency function, mode shape, concrete beam

## Abstract

In this study, tapered polymer fiber sensors (TPFSs) have been employed to detect the vibration of a reinforced concrete beam (RC beam). The sensing principle was based on transmission modes theory. The natural frequency of an RC beam was theoretically analyzed. Experiments were carried out with sensors mounted on the surface or embedded in the RC beam. Vibration detection results agreed well with Kistler accelerometers. The experimental results found that both the accelerometer and TPFS detected the natural frequency function of a vibrated RC beam well. The mode shapes of the RC beam were also found by using the TPFSs. The proposed vibration detection method provides a cost-comparable solution for a structural health monitoring (SHM) system in civil engineering.

## 1. Introduction

Structural Health Monitoring (SHM) is vital, not only in mechanical structures but also in concrete structures, and has received much attention in both researches and developments in recent years. In general, a typical SHM system comprises the integration of sensor techniques [1], smart materials, data interrogation and transmission, computational power, and processing ability inside the structures. Among these components in an SHM system, sensor technique plays an important role in monitoring not only the structural status—such as stress, displacement, acceleration, and vibration [2,3,4]—but also the influential environment parameters, for instance, wind speed, temperature, and others [5,6]. Reinforced concrete (RC) beam vibration detection provides tremendous information that indicates the health of a building. The natural frequencies and mode shapes have frequently been used for crack identification. The literature review found numerous research projects which have explored the vibration detection techniques. The work reported by Owolabi et al. has shown that the crack location and crack depths can be detected by the change in natural frequencies and mode shapes. It was found that knowing the crack position (prior to the crack size assessment) could result in accurate prediction of its extent in a crack identification problem if one uses only one mode. Besides, as the crack grows, the mode shapes undergo a highly noticeable change close to the crack location area. The changes in the natural frequency and mode shape depended on how close the crack was to nodes of mode shapes for higher modes [7]. 

Conventionally, the usage of sensors is mostly based on the transmission of electric signal, from which it inherits many disadvantages. For instance, the sensor heads are too big or not durable enough to measure interior properties when embedded in a structure. Electrical or magnetic interference (EMI) easily perturbs the transmission signal, and different demodulation techniques are required for different sensors [8]. Compared with conventional sensors, fiber optic sensors provide promising sensing in civil SHM systems. Fiber optic sensors are easily embedded into the host specimen, easily multiplexed and distributed, EMI-free, and have long life cycle [9]. Many fiber optic sensors have been proposed for applications in civil engineering SHM, especially for strain and vibration detection. 

Davis et al. have demonstrated that a multiplexed fiber Bragg grating (FBG) sensor array can be used to detect the dynamic strain of a cantilever beam, which is used to determine the shape and vibration mode [10]. A symmetrical pull–push structure was adopted to eliminate the FBG vibration sensor’s temperature drift [11]. Ling et al. proposed an embedded multiplexed FBG sensor to monitor the dynamic strain and vibration for intact and delaminated composite beams under various external excitations [12]. A dynamic strain calibration between FBGs and surface-mounted strain gauges was reported to find the correlation between strain and photovoltage. In the experiment, the monitoring result of FBG was compared with a laser vibrometer and an accelerometer. Experimental results revealed that embedded FBG has the ability to measure dynamic strain and identify the existence of delamination and other common damage in both mechanical and civil structures [13,14,15,16].

The FBG system provides high precision but inherits a large time delay and limited acquisition frequency, which makes it difficult for high frequency vibration detection. However, a multichannel FBG sensor system was proposed by Mueller et al. using a position-sensitive detector for wavelength interrogation [17]. A theoretical maximum bandwidth of over 300 kHz was achieved by using this approach, which is much higher than other systems. Such a system was tested for high-precision static strain measurement for shape control applications at low frequency in a test rip (<0.3 µm/m) at 5 Hz and more than 12 kHz for high-frequency dynamic strain measurement. 

Previously, polymer optical fiber (POF)-based sensors have been investigated extensively for many applications, such as crack detection in a concrete beam [18], curing process of cement paste [19], and so on. Conventionally, the intensity modulation technique is employed to demodulate the detected sensing signals. It is different from the FBG modulation technique; in an intensity modulation-based fiber sensor system it is easier to recover the detected sensing signals. In 2003, Kuang et al. reported the use of POF sensor and Ni–Ti shape memory alloy (SMA) wires as strain monitors of woven carbon fiber epoxy composite cantilever beams subjected to vibration tests [20]. SMA wires were applied to control and modify the damping response of the composite beam. The damping ratio did not change too much between 2 J and 4 J, suggesting that this sensing system is more suitable for larger impact energies. Kuang and Cantwell have demonstrated that the POF sensors have the ability to monitor the dynamic response of fiber composite beams [21]. The sensors were both surface-bonded to a plastic beam or embedded in a carbon-fiber composite, it was subjected to a series force and free vibration to assess this ability. The results of the natural frequencies of the beam agreed with theoretical values and strain gauge. The impact test experimental data also demonstrated that the POF sensors can be used to monitor the out-of-plane deflection during the impact event. Those results were validated by employing a laser Doppler velocimeter and piezoelectric load cell. An intensity modulation-based POF sensor was used to measure both static and dynamic strain on a cantilevered beam [22]. The strain change is detected by the transmitted light intensity. The POF sensors were fabricated using three types of housing tube (silicon rubber tube, rubber tube, and polytetrafluoroethylene (PTFE) tube) and their performances were evaluated separately. For a static test, the results showed that soft material provided better sensitivity than hard material (PTFE tube), however, the PTFE tube gave faster response time on dynamic strain. 

In this study, the performance of tapered polymer fiber sensors (TPFSs) for an RC beam vibration detection was explored for the first time. A multimode POF was chosen because of its typical inherent properties, such as better signal coupling, larger core radii, higher numerical aperture, and larger thermal-optic coefficient, compared to those of optical glass fiber. A tapering structure is created on the POF to enlarge its vibration detection sensitivity due to the effect of stress concentration, which makes the tapered section experience more stress than standard fiber from both sides. Besides, a low-cost multiplexing system was used to integrate many sensors into one system, which has the ability to modulate and demodulate the sensors. The performances of TPFSs were calibrated by an accelerometer. The TPFSs were surface-mounted and embedded in the RC beam. The natural frequencies and mode shapes of the RC beam were found by employing the TPFSs.

## 2. Sensing Principle and Beam Analysis

### 2.1. Sensing Principle

**Figure 1:**
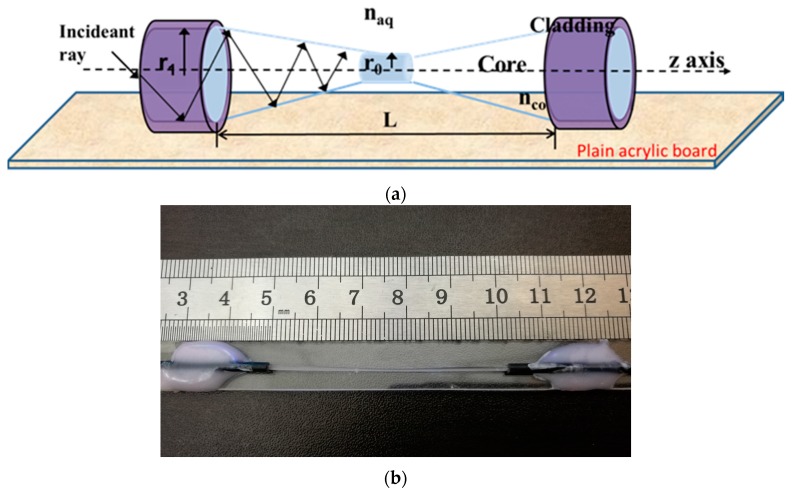
(**a**) Schematic diagram; (**b**) photograph of bonded tapered polymer fiber sensor (TPFS).

The configuration of a TPFS is shown in Figure 1. Figure 1 depicts ray propagation along the TPFS. The *z*-axis is the core axis of TPFS with an origin (*z* = 0) at the center of the taper structure. The ray propagation from taper length L/2 results in the radius of the tapered fiber decreasing from *r*_1_ to *r*_0_ along the tapered region. The refractive index of fiber core is *n_co_*, and the refractive index of ambient environment is *n_aq_*. The tapered fiber shows more potential in sensing elements and enables the ability to generate evanescent waves (EWs) associated with the change of propagating mode in optic fibers. When the TPFS is used for vibration detection, the tapered part is bent and induces a portion of guided light to become leak rays. The changing magnitude of the EW field detection can be measured by changing the output power in a tapered fiber-based sensor [23]. The vibration detection sensitivity can be enhanced significantly by tapering the fiber to a small diameter. This is mainly due to the effect of stress concentration (geometric discontinuities cause an object to experience a local increase in the intensity of a stress field), which makes the tapered section suffer more stress than a standard fiber from either side. Based on this principle, the TPFS was employed to detect vibration in the RC beam.

Removal of cladding results in a smaller refractive index in the sensing element than in the cladded region, where the cladding is replaced by air. This operation increased the number of propagation modes in the sensing element while the induction of fiber core results in the decrease of propagation modes. Thus, the V-number mismatch is encountered between the sensing element and cladded regions, therefore, the V-number in these two portions needs to be optimized for matching purpose. V-number is a parameter to describe the number of propagation modes guided by optic fiber, which is defined as

(1) $$V=\frac{2\pi r_{1}}{\lambda }\sqrt{n_{co}^{2}-n_{aq}^{2}}$$

where *λ* is the wavelength of transmitting light. For a bending fiber, the inner half experiences a compressive stress towards the bending center, and a tensile stress is applied along the outer half of the fiber. A conformal mapping technique can be employed to transform the curved fiber to an equivalent straight fiber when the TPFS is bent [21]

(2) $$V_{c}=\frac{2\pi r_{z}}{\lambda }\sqrt{n_{co2(\varepsilon )}^{2}-n_{aq}^{2}}$$

where $r_{z}=2z(r_{m}-r_{0})/L+r_{0}$ is the radius distribution of the linear fiber taper in the propagation direction of *z*, $r_{m}=r_{0}\sqrt{\left(n_{co2}^{2}-n_{cl}^{2})/(n_{co2}^{2}-n_{aq}^{2}\right)}$, taper ratio *T_r_* = (*r_z_* − *r*_0_)/*r*_0_, $n_{co2(\varepsilon )}=n_{co1(\varepsilon )}(1+R/n_{e})$, $n_{co1(\varepsilon )}=n_{co}+C_{f}S_{f}$, *C_f_* = −4.5 × 10^−12^ Pa^−1^ is the stress optic coefficient of fiber core, $S_{f}=\varepsilon_{f}E$ is the refractive index change of fiber core induced by the strain *ε_f_* applied on TPFS, *R* is the bending radius, $n_{e}=R/\left(1-n_{co1(\varepsilon )}^{2}(P_{12}-\nu (P_{11}+P_{12}))/2\right)$, *P*_11_ = 0.30 and *P*_12_ = 0.297 are the components of the photoelastic tensor, *E* = 0.1 × 10^10^ N/m^2^ is the Young’s modulus of POF, and ν = 0.34 is the Poisson’s ratio for POF. As shown in Equation (2), the numbers of transmission modes are determined by the tapered fiber radius *r*_0_, the refractive index of fiber core *n_co_*, and the refractive index of cement paste *n_aq_*. Hence, the total number of modes *M_T_* is given by

(3) $$M_{T}=\frac{V^{2}n_{co2}^{2}}{2(n_{co2}^{2}-n_{aq}^{2})}$$

**Figure 2:**
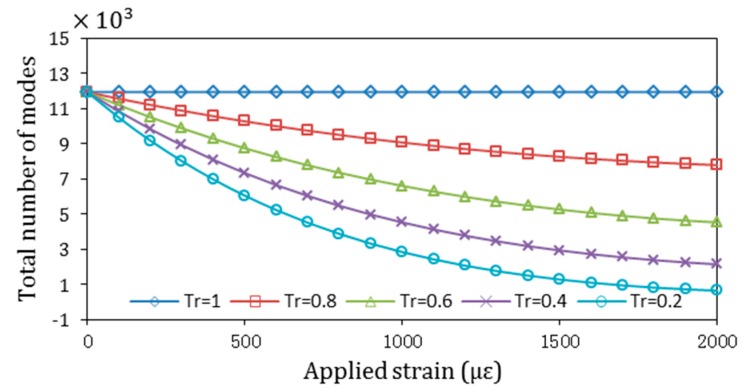
Total number of modes guided by the TPFS with various taper ratios and applied strains.

Total number of modes is a parameter that can be used to indicate the light intensity that is guided by TPFS. As such, the transmitted light intensity guided by TPFS can be modulated by the applied strain and thus its capability for vibration detection. The total number of modes guided by TPFS varied with the various taper ratios, and applied strains were numerically simulated (results are shown in Figure 2). The numerical results are based on the parameters given for plastic optical fiber: *r*_1_ = 480 μm, *n_co_* = 1.492, *n_cl_* = 1.402, *λ* = 594 nm, and *L* = 0.05 m. The results in this figure show that the total number of guided modes are decreased with the increased of applied strain. The decrease tendency of total number of modes for TPFS with smaller taper ratio is faster. The results also show that as the applied strain increases, the number of modes is decreased slightly for an untapered fiber (*T_r_* = 1). The large decrease tendency for the number of modes indicates the high vibration detection sensitivity of TPFS. Besides, the results in this figure indicated that the vibration detection sensitivity can be enhanced by tapering the POF significantly.

### 2.2. Beam Analysis

A single-degree-of-freedom (SDOF) system is used to describe the vibration of the RC beam, in which the RC beam can be treated as a simply supported beam. The motion can be described by a single coordinate, and the radian frequency depends on two system properties: mass and stiffness. Thus, for a simply supported beam, the equation of motion of Euler–Bernoulli is given by

(4) $$\frac{\partial^{2}y}{\partial t^{2}}+{\left(\frac{EI}{m}\right)}^{2}\frac{\partial^{4}y}{\partial x^{4}}=0$$

where *x* and *y* denote the distance along the beam from the support point and the displacement from the neutral axis, respectively, $E=57,000\times \sqrt{f_{c}}$ is the Young’s modulus of elasticity, *f_c_* is the concrete curing strength in the unit of *psi*, $I=\frac{a\times b^{3}}{12}$ is the moment of inertia, and *m* is mass. Using the boundary conditions of zero displacement and slope at the simply supported beam, along with the zero moment and shear force at the beam, the natural frequencies of vibration and shape of the beam can be found. Solving Equation (4), the natural frequency of the simple supported beam can be given as

(5) $$\omega_{n}^{2}=\beta_{n}^{4}\frac{EI}{\left(\rho A\right)}$$

where *ρ* is the density of the material, *A* is the cross-sectional area of the RC beam, *β_n_* is the boundary condition for different natural frequencies. For a simply supported beam, the boundary conditions are given by

(6) $$\beta_{n}l=n\times \pi$$

where *l* is the effective length of the RC beam and *n* is vibration mode of the beam. For the reinforced steel-bar concrete beam, however, density is changed to

(7) $$\rho =\frac{2A_{s}}{ab}\rho_{s}+(1-\frac{2A_{s}}{ab})\rho_{c}$$

where *ρ_s_* and *ρ_c_* are the density of steel bar and concrete, respectively, *A_s_* is the cross-sectional area of reinforced steel bar, and *a* and *b* are the width and height of the RC beam. The boundary conditions can also be used to determine the mode shapes from the solution for the displacement [24]

(8) $${\tilde{\omega }}_{n}=A_{1}\left[\left(\text{sin}\;\beta_{n}x+\text{sinh}\;\beta_{n}x)-\frac{\text{sin}\;\beta_{n}l-\text{sinh}\;\beta_{n}l}{\text{cos}\;\beta_{n}l-\text{cosh}\;\beta_{n}l}(\text{cos}\;\beta_{n}x+\text{cosh}\;\beta_{n}x\right)\right]$$

where *A*_1_ is a constant representing the magnitude of the vibration mode—typically a value of *A*_1_ = 1 is used when plotting mode shapes. Equation (8) can be used along with the strain information given by the TPFS attached to the RC beam for shape determination.

## 3. Experimental Materials and Methods

### 3.1. Sensor Probe Fabrication and Installations

The POF was purchased from Mitsubishi with fiber diameter of 1 mm, core diameter of 980 µm, and refractive indices of 1.492 and 1.402 for the core and cladding, respectively. To taper the fiber, acetone solution was applied to optical fiber by using cotton buds to dissolve some contents of polymer fiber. Then, the distilled water was used to neutralize the reaction between fiber and acetone. The milky white surface was carefully removed by using sandpaper with grit size of 320. This process was repeated until the required tapering length and diameter were formed. Finally, tapered optical fiber was cleaned by using the 2-propanol. By using this process, it is easy to control the geometrical structure of tapered fiber since the reaction of acetone and polymer fiber is uniform in the perform region. The fiber tip was cut by shears and polished by sandpaper carefully to make sure a vertical facet was formed. The tapered optical fiber with a tapered length of 5.0 cm and diameter of 480 µm was used in this study.

Figure 1 shows the configuration of bonded TPFS, of which two sides of TPFS were clamped and a constant pulling was applied to place the TPFS under stress. Fast-cure cyanoacrylate-based adhesive was used to glue two sides of TPFS onto a plain acrylic board. TPFSs were stored at room temperature for several hours to ensure the adhesive was cured, after which the applied stress was released. The output light intensity of TPFS was monitored during the aforementioned processes. Initially, the light intensity was slightly reduced as the TPFS was under-stressed. However, no significant change of light intensities was observed as a result of the bonding process. As shown in Figure 1, the tapered portion of TPFS is free of any residual adhesive and plain acrylic board, which would induce in an optically nonuniform distribution on the tapered area. 

**Figure 3:**
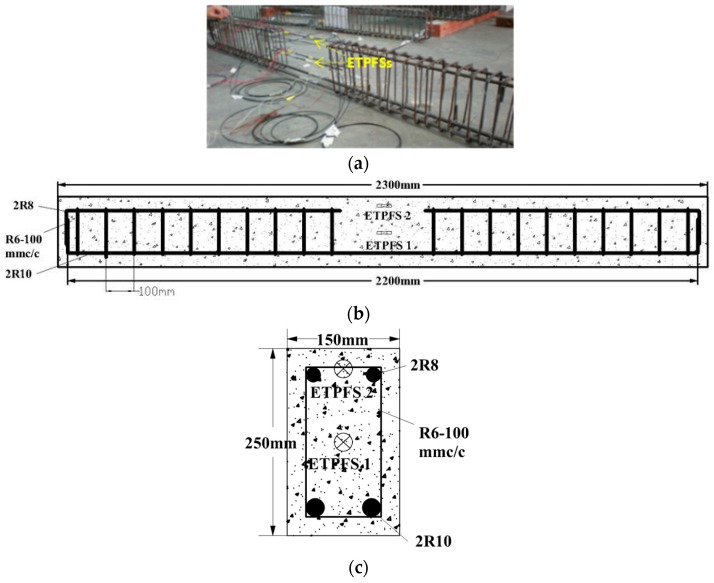
The reinforcement configuration of the reinforced concrete (RC) beam: (**a**) layout of reinforcements; (**b**) cross-section view of reinforcements and embedded TPFS (ETPFS); (**c**) photograph of configuration of reinforcements.

In the second experiment, the TPFSs were embedded into an RC beam as shown in Figure 3a. The TPFSs had prestressed bonding on the reinforcement steel bar before casting the RC beam. The transmission light intensity was detected during the bonding process and the RC beam casting process. A slightly reduced light intensity was observed as a result of the RC beam casting, however, it became stable after curing of the RC beam. For an embedded TPFS (ETPFS), the tapered portion was surrounded by concrete, which has a refractive index of 1.73 for the real part and 0.003 for the imaginary part [25,26]. A reasonable assumption can be made to guarantee the TPFS was only disturbed by the vibration: the refractive index change of cement paste induced by the vibration can be ignored. It is reasonable to make this assumption because the small change of refractive index of concrete only perturbs the higher-order modes that are guided by TPFS, in which the energy carried by the higher-order modes is much less than that of lower-order modes.

### 3.2. Reinforced Concrete Beam Specimen

Two reinforced RC beams with dimensions of 150 mm × 250 mm × 2300 mm were prepared using standard procedures. The details of the mix proportion of these beams are shown in Table 1. The cement grade of 30 was used in this experiment.

The configuration of reinforced RC beam was photographed and is shown in Figure 3b. A concrete strength of 40 MPa was cured for two reinforced RC beams, respectively. Three cylinder samples were casted, and materials used were the same with the RC beams. The loading test using the Universal Testing Machine (UTM; Instron 8800, INSTRON, Norwood, MA, USA) was performed after it fully cured. The results found that three samples had strengths of 39.2 MPa, 40.8 MPa, and 40.3 MPa. The image in Figure 3b shows that the reinforced RC beams consist of deformed bars with diameter of 10 mm, plain bars with diameter of 8 mm, and steel stirrups with diameter of 6 mm. Two TPFSs were embedded into an RC beam. The locations of embedded TPFS is shown in Figure 3c. One TPFS is mounted at the top surface of the RC beam, which withstands different strain with that of embedded in the center of RC beam. The natural frequencies of the RC beam can be calculated from Equation (5), which are 2.79 Hz, 11.2 Hz, and 25.1 Hz for *ρ_s_* = 7.9 × 10^3^/kg·m^−3^, *ρ_c_* = 2.43 × 10^3^/kg·m^−3^, *f_c_* = 5802 psi, *A_s_* = 0.0264 m^2^, *a* = 0.15 m, *b* = 0.25 m, and *l* = 2.3 m.

### 3.3. TPFS Multiplexing System

**Figure 4:**
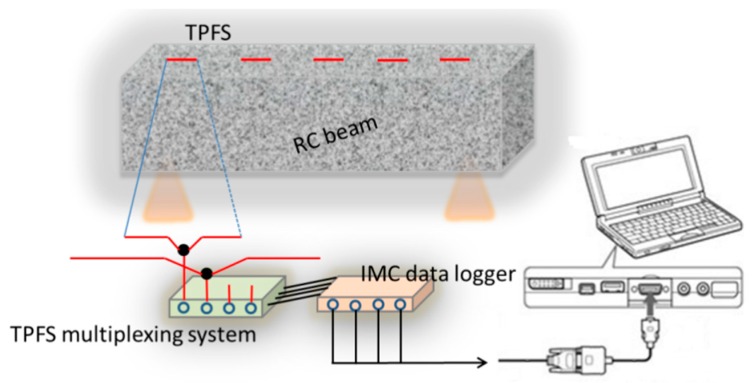
Experimental setup of TPFSs systems for the RC beam vibration test.

The TPFS multiplexing system for vibration and loading test monitoring is shown in Figure 4. In this system, four sensor probes are multiplexed into one system which integrates four the light-emitting diodes (LEDs) and four Photo detectors (PDs) to emit and detect the light, respectively. Five sensor probes were produced using the POF with the same parameters. A circuit was designed to provide a stable current to drive LED (red light, centered at 660 nm) and PDs. The LEDs driven by this circuit show an excellent stability for output light intensity. The PD converts the light power into voltage signal. Then, the voltage signals are received by an integrated measurement control instrumentation (IMC) data logger. A long-term drift study indicates that the driving circuit had a very good stability, which is ±2%. In this system, all of the TPFSs are synchronized to detect the vibration on a reinforced RC beam. The room temperature was also monitored by a thermocouple. The test was performed for 48 h, and a maximum drift of ~2% and 2.0 °C was found for light intensity and room temperature, respectively. This is indicating that the experimental works are under a very well-controlled ambient condition.

### 3.4. Vibration Monitoring System

**Figure 5:**
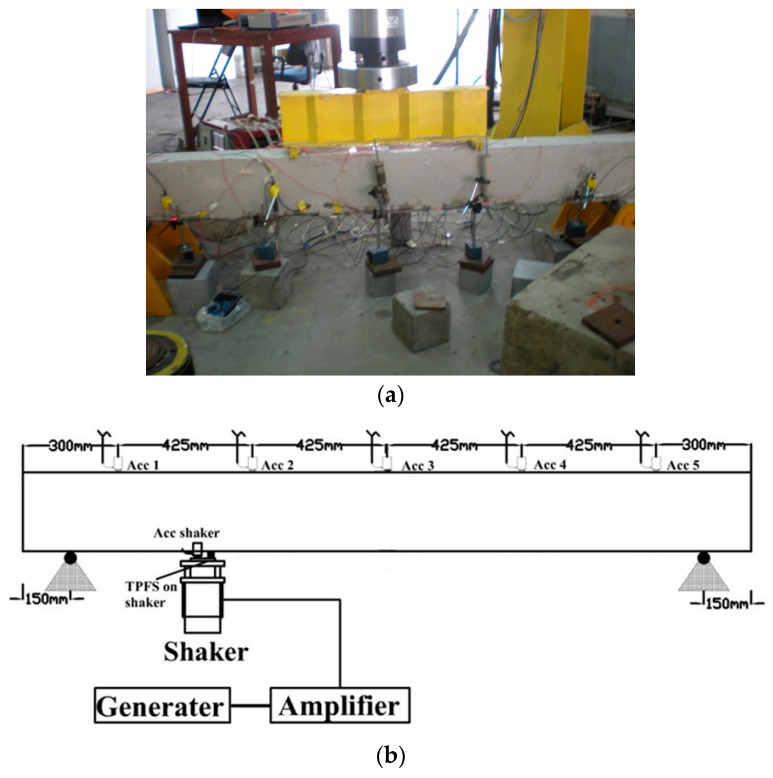

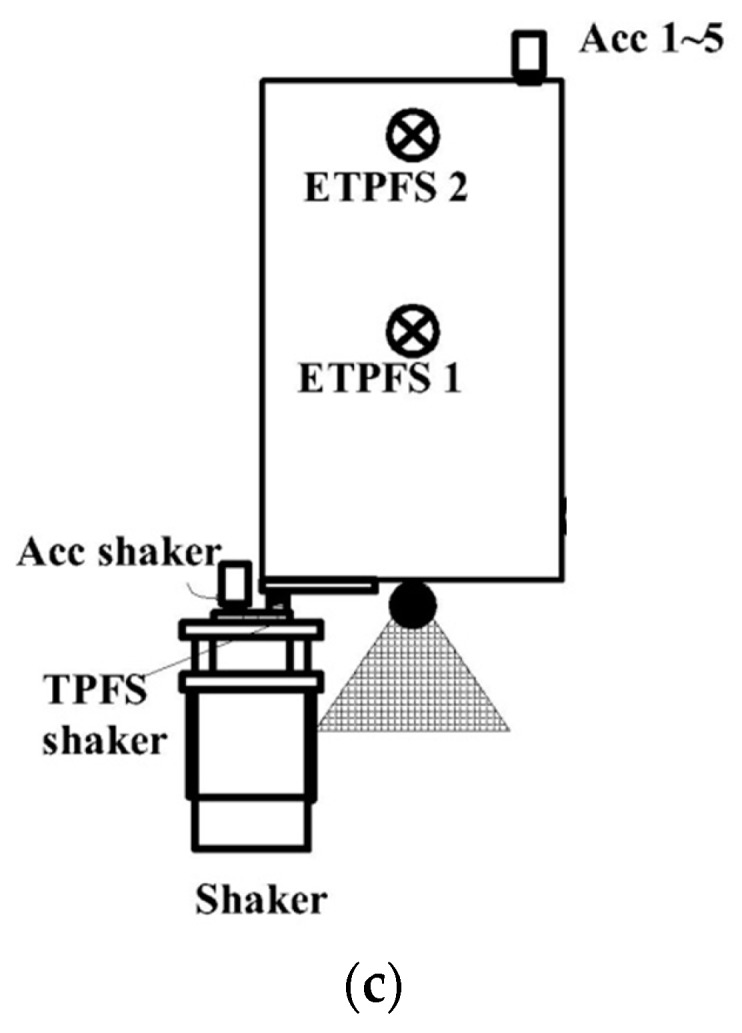
Experimental setup of vibration monitoring system: (**a**) photograph of vibration test for the RC beam; (**b**) layout of accelerometers, surface-mounted TPFSs (SMTPFSs) and ETPFSs installation; (**c**) cross-section view of sensors installation.

The configuration of the vibration monitoring system is shown in Figure 5. A 3.1 MHz function generator (model D5335, SRS (Stanford Research System), Sunnyvale, CA, USA) was set at frequency range of 1–100 Hz to produce a fast sine sweep signal. The signal was then amplified by an amplifier to drive the shaker, which eventually transfers the force to the RC beam. To avoid the RC beam from responding to the excitation, the shaker was placed as shown in Figure 5b, which is far away from a nodal point. As shown in this figure, a total of six Kistler accelerometers (KISTLER, Amherst, NY, USA) were used as reference devices for measurement of the dynamic response of the RC beam. Five were attached on the upper surface of the RC beam, and one of them and a TPFS were mounted on the surface of the shaker as a reference input. Five TPFSs were mounted on the surface of the RC beam after completing the vibration experiment of accelerometers, which were located at the same position with that of Kistler accelerometer. Each sensor was positioned at a distance of 425 mm apart. The sensors were attached on the RC beam to provide the information of the vibration of the RC beam while the mounted sensors on the shaker were used to provide the reference information. Besides, two ETPFSs were employed to monitor the vibration of the RC beam as shown in Figure 5b. An IMC data logger (model imc CRONOSflex, imc Test & Measurement, Friedrichsdorf, Germany) was used to collect the signals of Kistler accelerometers and TPFSs, respectively. The IMC data logger was set to acquire at 100 Hz. The system with this setting provides a very high precision for vibration detection for both Kistler accelerometers and TPFSs. An ME’scope software (Vibrant Technology, Centennial, CO, USA) was used for data analysis to determine the frequency responses and mode shapes of the RC beam. The first three natural frequencies and mode shapes of the RC beam were identified.

## 4. Results and Discussion

### 4.1. Calibration the TPFS

**Figure 6:**
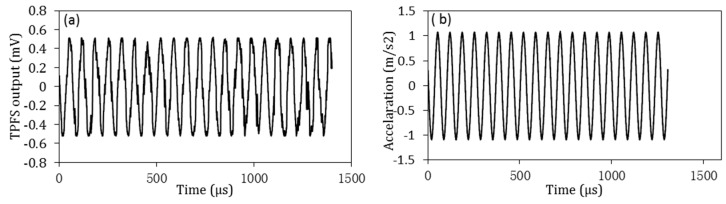
(**a**) Responses of TPFS; and (**b**) accelerometer for vibration frequency of 30Hz.

**Figure 7:**
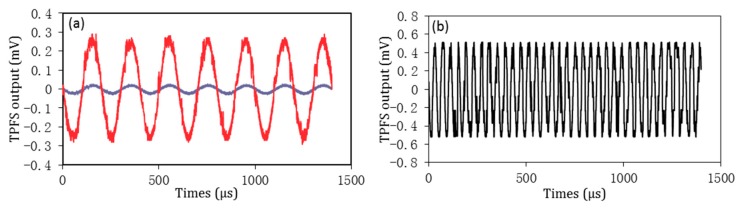
(**a**) Comparison the response of TPFS for frequency at 10 Hz with different vibration amplitude; (**b**) the response of TPFS for frequency of 50 Hz.

Before the vibration detection of the RC beam, the accelerometer and TPFS was calibrated on a small-precision vibration table, which was excited by a vibration control system including function generator, filter, and amplifier. The responses of TPFS and accelerometer are shown in Figure 6, when the vibration table was excited by a sine wave with frequency of 30 Hz. From the results in Figure 6, it can be observed that both the TPFS and accelerometer have responded well to excited wave. It proves that the proposed TPFS can be used to detect the vibration. Furthermore, the TPFS was also employed to test the response for vibration frequency of 10 Hz with different excited amplitudes of 0.2 G and 1 G, and the results are shown in Figure 7a. As shown in this figure, the output voltage of TPFS varies with the change of amplitude of excited wave. A well-respond wave is shown in Figure 7b, which proved that the TPFS has ability to detect the vibration frequency up to 50 Hz. 

### 4.2. Comparison the Performances on the RC Beam

**Figure 8:**
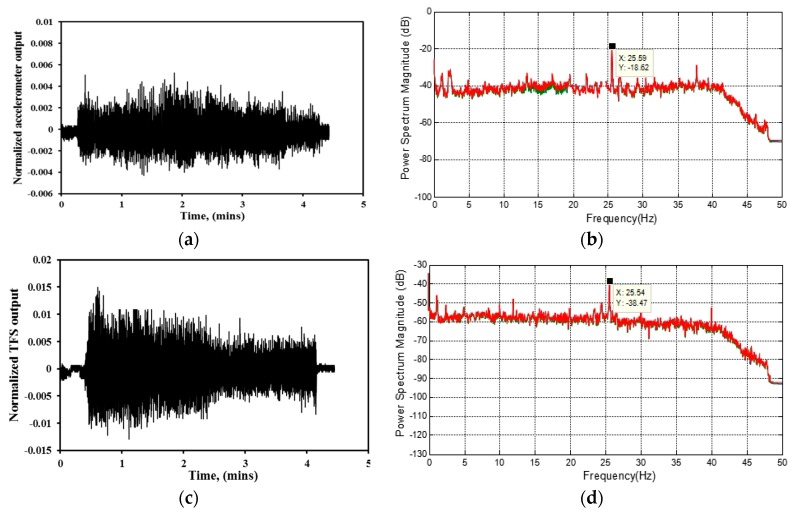

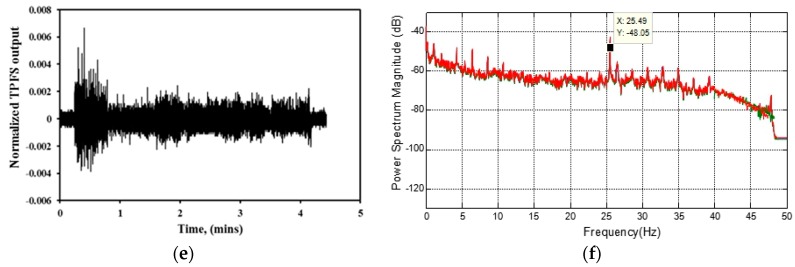
Detected vibration in time domains and their corresponding frequency domains on the RC beam for (**a**,**b**) Kistler accelerometer; (**c**,**d**) ETPFS; (**e**,**f**) SMTPFS.

The vibration test was implemented first by employing the accelerometers and TPFSs to compare their performances. The beam was excited by a shaker in the frequency range of 1–100 Hz with sweep rate of 0.01 Hz. The vibration detection results based on accelerometer and TPFSs were employed to establish the change in modal frequencies of the RC beam. For a given structure, the damage detection methods based on modal frequencies are that any shifts in the natural frequencies indicate a change in its structure properties has taken place. The raw data detected by accelerometers and TPFSs were stored in a time domain. In order to see its frequency responses, ME’scope software functioned with a low-pass Butterworth filter was applied to remove its high frequency parts, then, a fast Fourier transform (FFT) was performed for all of the data. By employing this technique, the raw data were transformed into the frequency domain to show the frequency response function. The natural frequencies are global and can be observed from frequency response function, which can be taken from virtually any point on the beam. The results in Figure 8 are the raw data in time domains and their corresponding frequency domains. The results detected by other accelerometers and TPFSs are similar to the results presented here and they are not shown. From Figure 8, it can be observed that both the accelerometer and TPFSs have the ability to indicate the vibration of the RC beam. From Figure 8 a,c,e, it can be observed that the responses of TPFSs are constant with that of the accelerometer. Both can precisely reflect the time for vibration start and stop. From Figure 8b,d,f, it can be seen that both have several spectrum magnitude peaks, and each of these peaks show the same trends and location. It also can be observed that the amplitude of frequency response function for ETPFS is higher than SMTPFS. This may be due to the ETPFS being surrounded by cement, which may withstand more energy from excitation.

### 4.3. Vibration Detection of RC Beam

**Figure 9:**
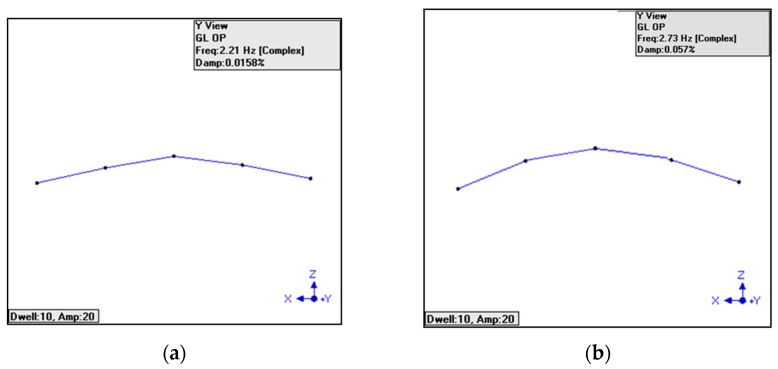

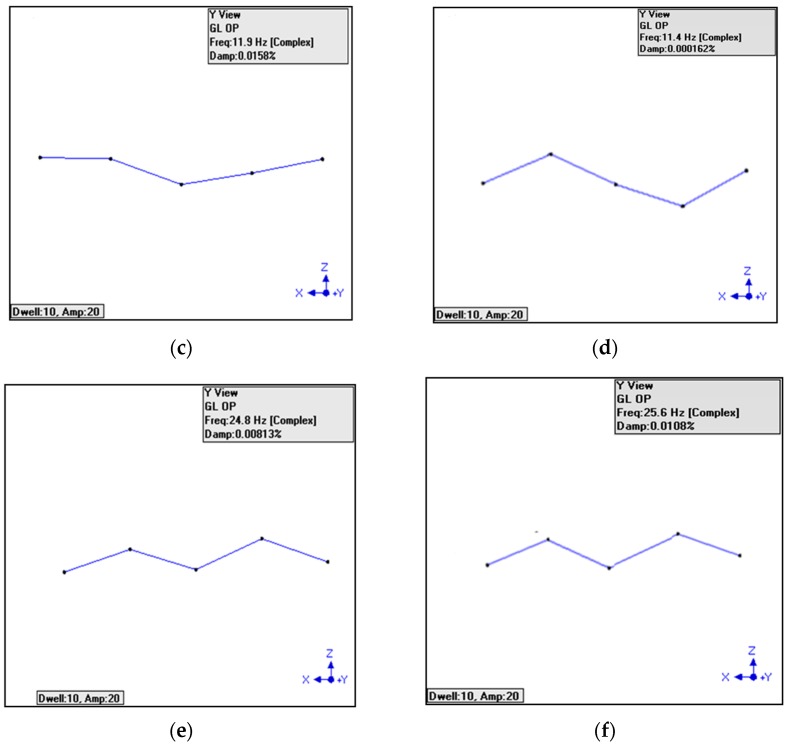
Mode shapes of the RC beam detected by SMTPFSs for (**a**) mode 1; (**c**) mode 2; (**e**) mode 3; and by accelerometers for (**b**) mode 1; (**d**) mode 2; (**f**) almode 3.

The natural frequencies and mode shapes were obtained from five SMTPFSs and accelerometers, respectively, and their results are shown in Figure 9. The natural frequencies obtained from TPFSs, accelerometers, and numerical results are summarized in Table 2. The natural frequencies of the RC beam when utilizing TPFSs and accelerometers have been detected respectively with the values of 2.21 Hz, 11.9 Hz, 24.8 Hz; and 2.73 Hz, 11.4 Hz, 25.6 Hz. From this table, it can be found that the differences between the numerical results and accelerometers are smaller for the first order and second order of natural frequencies. However, the difference for third-order natural frequency is small between TPFSs and numerical results. The detected mode shapes of the RC beam are also given in Figure 9. From these figures, we can see that the fitted curves of three modes are well-detected by TPFSs and accelerometers for a simply support RC beam. However, the result shown in Figure 9c is slightly different from that of in Figure 9d. Hence, a nearly perfect mode shape is desired for using the TPFSs, and performance needs to be optimized in future. As such, from the results showed in Figure 9, we can conclude that the proposed TPFS has good ability to detect the natural frequency and mode shape to indicate the vibration of the RC beam, which are the signs to evaluate the health condition of the RC beam.

## 5. Conclusions

In summary, the TPFSs have demonstrated vibration detection ability on the RC beams. A V-number theory was employed to show the sensing principle of the proposed sensor. Numerical results show that V-number decreases with the increase of applied strain. A low-cost integrated monitoring system was built, in which many TPFSs were multiplexing. For vibration detection, the responses of TPFSs were calibrated with accelerometers. Two different installation methods of TPFSs were studied and experimentally demonstrated. Experimental results show that the amplitude of frequency response function for ETPFS is higher than SMTPFS. This study proves that the installation of the TPFSs is flexible for civil structures. Five SMTPFSs were used in the experiment. The results showed that TPFSs have the ability to detect the natural frequency and mode shape of the RC beam. The characteristics of TPFSs, such as high mechanical strength, make it easily embedded into the host specimen and an ideal alternative for the applications of the structural health monitoring in the civil engineering.

## Figures and Tables

**Table 1:** The mixture of cementitious materials for reinforced RC beams.

Materials per m^3^	Ordinary Portland Cement (OPC) (kg)	Water (kg)	Coarse Aggregate (kg)	Sand (kg)
w/c = 0.67	317	208	735	1140

**Table 2:** Natural frequencies of the RC beam obtained from TPFSs, accelerometers, and numerical results.

Natural Frequencies (Hz)	Numerical Results	Accelerometers	TPFSs
First order	2.79	2.73	2.21
Second order	11.2	11.4	11.9
Third order	25.1	25.6	24.8
